# The Ins and Outs of Antigen Uptake in B cells

**DOI:** 10.3389/fimmu.2022.892169

**Published:** 2022-04-26

**Authors:** Adam Nathan McShane, Dessislava Malinova

**Affiliations:** Wellcome-Wolfson Institute for Experimental Medicine, Queen’s University Belfast, Belfast, United Kingdom

**Keywords:** B cell receptor (BCR), endocytosis, antigen uptake, antigen presentation, clathrin, endophilin A2, caveolae, phagocytosis

## Abstract

A review of our current knowledge of B cell antigen uptake mechanisms, the relevance of these processes to pathology, and outstanding questions in the field. Specific antigens induce B cell activation through the B cell receptor (BCR) which initiates downstream signaling and undergoes endocytosis. While extensive research has shed light on the signaling pathways in health and disease, the endocytic mechanisms remain largely uncharacterized. Given the importance of BCR-antigen internalization for antigen presentation in initiating adaptive immune responses and its role in autoimmunity and malignancy, understanding the molecular mechanisms represents critical, and largely untapped, potential therapeutics. In this review, we discuss recent advancements in our understanding of BCR endocytic mechanisms and the role of the actin cytoskeleton and post-translational modifications in regulating BCR uptake. We discuss dysregulated BCR endocytosis in the context of B cell malignancies and autoimmune disorders. Finally, we pose several outstanding mechanistic questions which will critically advance our understanding of the coordination between BCR endocytosis and B cell activation.

## Introduction

The term endocytosis describes the production of intracellular membranes from the cell surface plasma membrane (PM), a phospholipid bilayer, to internalize extracellular components, such as cell surface receptors and soluble extracellular factors. Once internalized, the contents can be recycled back to the PM through recycling pathways or delivered to late endosomes and lysosomes for cellular degradation. The final destination of internalized cargo is dependent on post-translational modifications (PTM) and interactions with distinct trafficking adaptors (1). Endocytosis is a ubiquitous cellular process and cargo includes receptors for iron uptake, cytokine responses, growth factors, glucose, cholesterol, vitamins (2–4). As such, it plays a critical role in cell growth and metabolism, cell-cell interactions, adhesion, and migration.

In the immune system, multiple endocytic pathways, including phagocytosis, pinocytosis, receptor-mediated endocytosis and caveolae, allow the uptake of foreign antigens for processing and presentation, and subsequent initiation of immune responses. Different antigen presenting cells rely on distinct endocytic mechanisms; dendritic cells (DC) have the structural and membrane capacity for dramatic and energetically demanding forms of endocytosis such as macropinocytosis and phagocytosis of particulate antigen. In contrast, the size and membrane availability in B cells, together with their requirement to detect specific antigens, limits their antigen uptake capacity to receptor-mediated endocytosis. Soluble antigens encountered in the lymphatics or membrane-bound antigen displayed by antigen presenting cells (APC) (5), binds to the surface B cell receptor (BCR), which initiates downstream signaling cascades for B cell activation and rapid endocytosis to provide material for antigen presentation. In this review, we will discuss mechanisms of antigen-BCR endocytosis in B cells, their coordination with downstream BCR signaling and their dysregulation contributing to B cell malignancy and autoimmunity.

### B Cell Activation

B cells promote clearance of invading pathogens and long-term immunity through two main cellular processes: antigen presentation on major histocompatibility complex class II (MHC-II) molecules to initiate T cell responses (6); and differentiation into memory or plasma cells for specific antibody production. Both processes require the coordination of signaling and endocytosis at the BCR.

The BCR is composed of surface immunoglobulin (Ig) and non-covalently associated Igα/β heterodimer. Igα and Igβ chains possess a cytoplasmic tail containing immunoreceptor tyrosine-based activation motifs (ITAMS), which allow signal transduction. Upon antigen binding, ITAMs can be phosphorylated to transmit downstream signals through the action of several enzymes (such as Src family kinase Lyn), reviewed in detail elsewhere (7).

In addition to signaling, antigen binding triggers BCR endocytosis and trafficking of the BCR-antigen complex into antigen-processing compartments, where antigenic peptides are loaded onto MHC-II. Presentation of antigen-loaded MHC-II molecules on the B cell surface promotes interaction with cognate CD4+ T cells, which stimulates B cell activation and transcriptional changes. Thus, BCR-mediated antigen internalization and processing determines the number and repertoire of antigen peptides presented, directly impacting B cell ability to compete for T cell help during the germinal center (GC) response (8).

Despite this interconnectivity, little is known about how BCR signaling and endocytosis are coordinated. Indeed, the two processes appear to be mutually exclusive for individual BCRs as the phosphorylation of ITAMs required to initiate signaling, is incompatible with binding of known endocytic adaptors (9, 10).

### Molecular Mechanisms of BCR Internalization

#### Clathrin-Mediated Endocytosis

The best characterized mechanism of BCR internalization is clathrin-mediated endocytosis (CME) (11, 12). Upon antigen binding to the BCR, clathrin is recruited to BCR-antigen clusters *via* its essential adaptor protein-2 (AP-2), which binds ITAM residues in the cytoplasmic tails of Igα and Igβ (10). BCR-proximal Src family kinases mediate phosphorylation of clathrin heavy chain, promoting binding to clathrin light chain and polymerization to induce membrane-curvature and formation of clathrin-coated pits (CCP) (13–15). Clathrin heavy chain phosphorylation also promotes association of CCPs with the actin cytoskeleton (14, 16). BCR antigen binding also promotes tyrosine phosphorylation of several CCP-associated proteins, including Epsin15 and Intersectin2 (17, 18), further cementing the link between BCR signaling and CME. CCPs invaginate before scission from the PM mediated by the large GTPase dynamin. Dynamin forms a helical polymer around the constricted neck of the CCP and, upon hydrolysis of GTP, mediates membrane scission producing clathrin-coated vesicles (CCV). This mechanically demanding process requires actin polymerization which may be mediated by interaction between clathrin light chain and its actin-binding partner Hip1R (16, 19). The specific clathrin adaptors, redundancy, stoichiometry and dynamics may be altered depending on antigen concentration (20).

**Figure 1:**
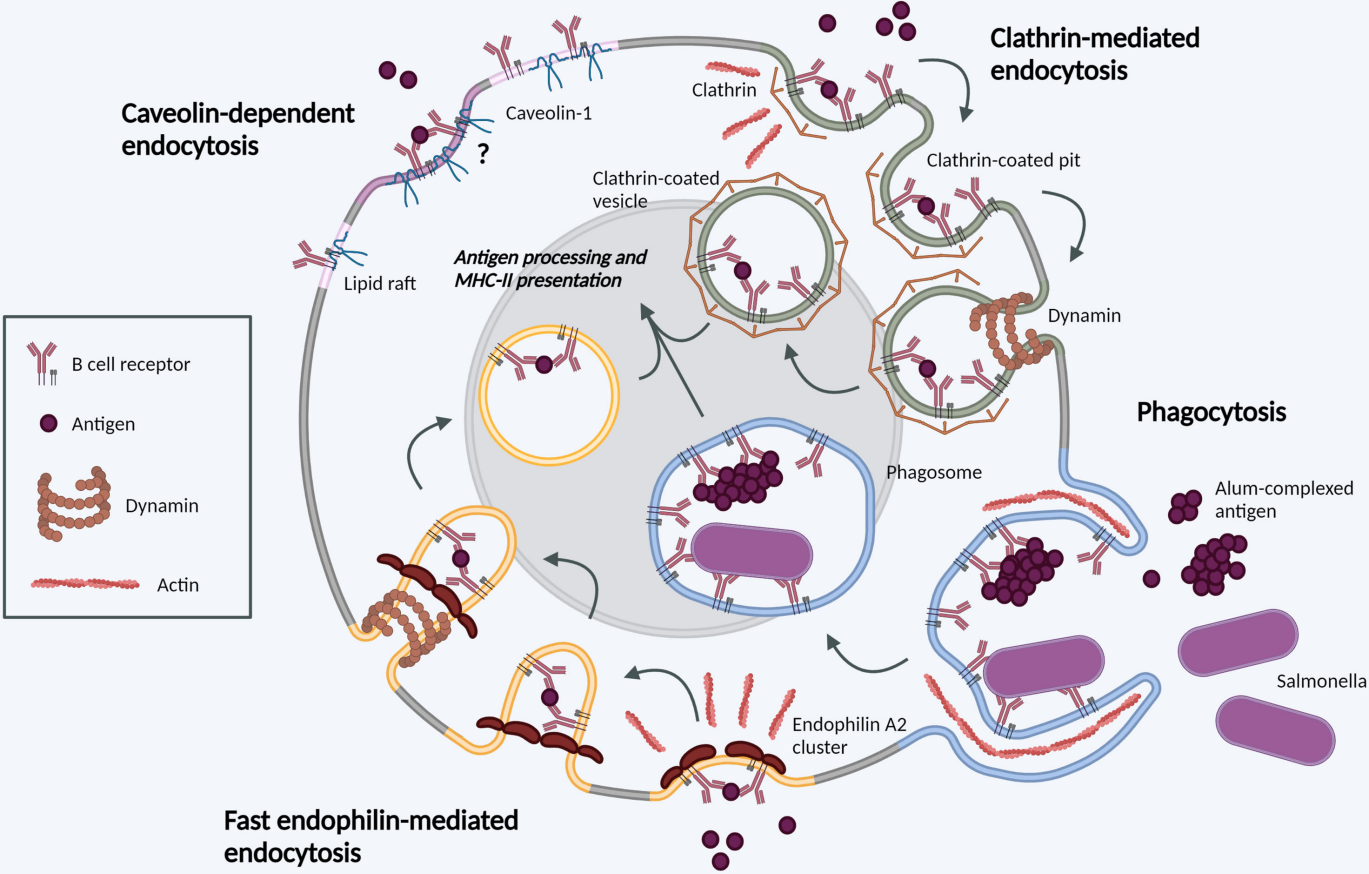
Molecular mechanisms of BCR internalization. Clathrin-mediated endocytosis (green). Following BCR antigen binding, clathrin is recruited to BCR-antigen clusters, binding ITAM residues in the cytoplasmic tails of Igα and Igβ. Clathrin polymerization induces membrane-curvature and formation of CCPs. CCPs invaginate before scission from the PM mediated by dynamin, forming a CCV. CCVs containing BCR-antigen complexes fuse with late endosomes for antigen processing and subsequent presentation. Phagocytosis (blue). BCR binding to large, particulate antigens can induce local actin rearrangements, driving membrane protrusion to form a large membrane invagination. Once internalized in phagosomes, the membrane vesicle undergoes fusion events in parallel with other endocytic processes. Fast endophilin-mediated endocytosis (yellow). Within endophilin-primed membrane patches, BCR antigen binding results in endophilin-mediated membrane curvature with subsequent dynamin scission to form an intracellular vesicle. This pathway is specific for ligand-triggered, signaling receptors. Caveolin-dependent endocytosis (pink). Caveolin-1 controls the distribution of surface BCR into lipid rafts. Its role in antigen-mediated BCR internalization requires further investigation. Created with BioRender.com.

Experimental inhibition of CME has revealed significant plasticity in BCR internalization pathways (21). This may be particularly striking in GC B cells, which do not polarize several CME components (e.g. sorting nexins SNX9, SNX18) to the immunological synapse (22) and appear more reliant on clathrin-independent endocytosis (23). We discuss current knowledge of these alternative pathways below (and **Figure 1**). 

#### Fast Endophilin-Mediated Endocytosis

FEME was described in 2014 as a fast-acting, tubulovesicular, clathrin-independent endocytic pathway. It mediates ligand-triggered uptake of several G-protein coupled receptors and receptor tyrosine kinases in a dynamin-dependent manner (24). FEME is mediated by BAR domain-containing endophilin proteins – Endophilin A1, A2 and A3. While Endophilin A1 and A3 are restricted to tissues such as brain, kidney, and testes, Endophilin A2 is ubiquitously expressed and importantly, the only endophilin family member expressed in B cells.

Endophilin A2 is present in PM patches, priming the membrane for FEME by sporadically forming and dissolving clusters. These clusters are highly dynamic, lasting 5-15s, and rely on production of PI (3, 4)P_2_ at the membrane (25). Within these clusters, productive receptor-ligand interactions result in cargo capture, endophilin-mediated membrane curvature and rapid endocytosis with subsequent dynamin-dependent scission. In contrast, in the absence of ligand binding, the membrane priming complex is dissembled and new endophilin clusters can form stochastically (26). The molecular mechanisms of FEME, and known or suspected cargo proteins in other cells have been extensively reviewed (25). Critically for B cells, unlike other pathways, FEME is non-constitutive ligand-triggered endocytosis.

Recent work identified the BCR as cargo for FEME. An unbiased whole-genome screen in a human B cell line identified a role for Endophilin A2 specifically in ligand-triggered BCR internalization. Although the stoichiometry and direct interactors remain to be discovered, recruitment of Endophilin A2 to the BCR was dependent on Grb2 and BLNK. Endophilin A2 deletion caused B cell-intrinsic defects resulting in decreased antigen-specific GC responses and reduced high-affinity IgG production (23).

Unlike CME, FEME critically requires Cdc42 activity and actin polymerization, concurrent with observations of BCR-antigen uptake in B cells. The use of FEME as an additional endocytic mechanism in B cell antigen internalization may guarantee internalization of phosphorylated BCRs, where machinery for signaling and clathrin-dependent endocytosis would normally compete for binding. In the context of other receptors, endophilin has also been described to interact with the CIN85-Cbl complex (27, 28), as well as ESCRT (endosomal sorting complexes required for transport) components ALIX and Tsg101 (29), which are highly expressed in germinal center B cells. Investigation of these interactions in B cells will advance our understanding of the coordination between BCR signaling and endocytosis. Further mechanistic insights will allow define the contribution of this pathway to antigen affinity discrimination, the dynamics and repertoire of antigen presentation, and its involvement in malignancy, given its specific requirement in Germinal Center B-cell like (GCB) and Burkitt lymphomas (23).

#### Caveolin-Dependent Endocytosis

Caveolin-1 is an integral membrane protein with a hairpin-like structure containing an internal intramembrane domain and both C- and N-termini facing the cytosol. Caveolin-1 promotes PM compartmentalization and the assembly of ordered lipid domains, such as caveolae - flask-shaped PM invaginations, with a diameter of 50-100nm, considered a form of lipid rafts. Early studies reported lack of expression of caveolin-1 in transformed B cells lines (30); however, subsequent work in primary cells has shown caveolin-1 expression in mouse and human B cells (31, 32). More recently, caveolin-1 was shown to control the distribution of IgM BCR clusters to ordered lipid domains on the B cell surface. Caveolin-1 function in BCR organization is dependent on Tyr14 phosphorylation by Src family kinases. In caveolin-1-deficient B cells, altered BCR nanoscale organization resulted in impaired BCR signaling. In immature B cells, loss of caveolin-1 resulted in defective central tolerance and expression of autoreactive BCRs leading to autoimmunity in mice (33). Interestingly, in these studies BCR internalization kinetics following soluble anti-IgM/IgD stimulation were unchanged in caveolin-1-deficient B cells. Questions remain regarding the mechanisms of caveolin-1 class-specific interactions with IgM or IgD BCR, and the potential role of caveolin-1 in cognate antigen uptake.

#### Phagocytosis

Phagocytosis is defined as the receptor-mediated ingestion of large (≥0.5-μm) particles through local rearrangements of the actin cytoskeleton. Irrespective of the surface receptor involved, this type of internalization results in a membrane-bound vacuole termed a phagosome, which matures through regulated fusion events, altering its composition and pH, in parallel with other endocytic pathways (34). Rho family GTPases, as master regulators of actin dynamics, play an essential role in PM and cytoskeleton remodeling during particle internalization (35, 36). The early paradigm dictated that phagocytosis was carried out by professional phagocytes, such as macrophages and DCs, providing APCs with antigen for presentation to lymphoid cells, and therefore playing a key role in the initiation of adaptive immune responses (36). B cells were thought to lack such phagocytic properties, due to the dramatic membrane and cytosolic rearrangements required, and have been used as negative controls in phagocytic assays (37). The discovery of macrophage-like B cell phagocytosis in a primitive vertebrate species transformed our understanding of the process (38). Subsequent studies identified BCR-mediated phagocytosis of bacteria in human B cells and murine innate-like B1 B cells, inducing robust antigen presentation and downstream B cell responses (39–41).

More recently, Martínez-Riaño et al. demonstrated that follicular B cell phagocytosis is BCR-driven and dependent on RhoG GTPase. This phagocytosis was important for development of a strong GC response and generation of high-affinity class-switched antibodies. Phagocytosis of antigen complexed to alum (common immunization adjuvant) provides a mechanism for initiating a humoral response against particulate antigens (42). The discovery that B cells phagocytose may play a pivotal role in vaccine design.

#### Trogocytosis

Trogocytosis is a process commonly described as cell gnawing, in which lymphocytes can internalize cellular material, including transfer of plasma membrane, from conjugated APCs. The process has been demonstrated in primary B cells and requires engagement of the BCR (43, 44) though may exhibit distinct signaling and cytoskeletal requirements from other cell types (45, 46). Most recently, trogocytosis was shown to mediate transfer of peptide-MHC II complexes from conventional DCs to marginal zone B cells in a complement receptor-dependent manner (47). This significantly expands the variety of antigens that marginal zone B cells can present to T cells, and opens further research avenues on the mechanisms and dynamics of peptide acquisition.

#### Endocytosis of Immobilized Antigens Following Proteolytic Extraction

A distinct mechanism of antigen endocytosis has been demonstrated for antigens presented on rigid substrates. In this model, B cell polarization towards the IS results in release of lysosomal content into the extracellular space (48, 49). Subsequent studies have revealed a role for Galectin8, a glycan-binding protein mediating extracellular matrix interactions, in B cell polarization, lysosome docking at the antigen contact site, and acidification of the IS space (50). Recent work provided further insight on lysosomal recruitment mechanisms. First, proteasome activity can promote actin clearance at the IS and lysosomal polarization towards immobilized antigens (51, 52). Second, interaction with surface-tethered high-affinity antigen induces B cell permeabilization and plasma membrane repair through calcium-induced lysosome exocytosis (53). Whether the specific endocytic mechanisms involved in recovery of proteolytically extracted antigen differ from those described above remains to be investigated.

### The Role of Actin in BCR Endocytosis and Signaling

In B cells, actin plays a crucial role in antigen acquisition in several distinct stages. First, cortical actin reorganization coordinates B cell spreading and contraction at the immunological synapse (54–56). Second, actin modulates spatial patterning of the surface BCR, regulating the amplitude/strength and dynamics of clustering and signaling by the BCR (57–59). Third, actin interacts with endocytic processes for antigen extraction and internalization. The first two stages have been extensively reviewed elsewhere (60–62) and here we will focus on the interaction with endocytosis. Actin foci have been described at sites of antigen uptake in murine and human B cells (22, 63, 64) and generate the physical force required to extract membrane-bound antigen (15). Deletion of the actin motor protein myosin IIa significantly reduced antigen uptake in naïve B cells (65), further highlighting the requirement for mechanical force.

In CME, Lyn-mediated phosphorylation of clathrin heavy chain promotes interaction with actin (14, 16). Antigen-induced BCR signaling through Vav, Bam32 and Btk can also regulate actin polymerization and association with CCPs (66–68). In addition, several clathrin-interacting proteins (CYFIP1, Intersectin, Eps15) can promote actin polymerization through Wiskott-Aldrich Syndrome protein (WASp) family proteins (69) and B cell deficient in WASp exhibit reduced antigen uptake (70). Actin is crucially required for antigen extraction by GC B cells, which apply stronger physical forces and exhibit distinct synaptic architecture (22, 71, 72). Investigation of individual clathrin adaptors and actin regulators will likely reveal further molecular details and differential requirements; for example, deletion of Itsn2, a Cdc42 guanine nucleotide exchange factor (GEF), reduced germinal center formation but did not impact BCR-antigen uptake (73). This highlights the challenges in investigating direct and indirect effects in this process.

Actin polymerization is also required for all clathrin-independent pathways we describe in B cells. In FEME, actin polymerization drives local membrane deformation and formation of intracellular bud (25, 74). Molecular details remain to be investigated but FEME requires all three main small GTPases (RhoA, Rac1 and Cdc42), with Cdc42 required for priming and inhibition of RhoA or Rac1 inhibiting FEME entirely (24). Phagocytosis of larger antigens also requires intense remodeling of the actin cytoskeleton (42). Whether similar mechanisms are required for the poorly understood process of trogocytosis (45) remains to be investigated.

Although originally described to promote CME (14), lipid rafts (cholesterol-rich microdomains) have been shown to drive BCR internalization independently of clathrin (21). This uptake requires the immunoglobulin cytoplasmic domain (75) and is strongly dependent on intact actin remodeling (21). Caveolae are closely associated with actin filaments, through potential interactions with filaminA and Intersectin2L (76, 77). Dissociation of lipid rafts from the underlying cortical actin, through ezrin dephosphorylation and release, is critical for antigen-induced lipid raft coalescence and eventual uptake (78). Rapid cholesterol-mediated BCR uptake may explain uncoupled BCR signaling in anergic B cells (79). Whether this is related to reduced NFκB signaling observed in GC B cells (71) and, more broadly, the role of lipid rafts in GC antigen uptake requires further investigation. How lipid order in these microdomains directs protein interactions and whether rafts play a role in force-dependent affinity discrimination is unclear.

### Post-Translational Modifications of BCR-Associated Endocytic Proteins

Common PTMs downstream of BCR engagement include phosphorylation and ubiquitination. As described above, phosphorylation by proximal kinases not only initiates downstream signaling cascades but is required for interaction with numerous endocytic regulators (70). Its involvement in several pathways is not well understood, for example, Endophilin A2 is itself phosphorylated at serine 288 and the level of modification is marginally increased upon antigen challenge (18), however, the significance of this for FEME or CME is unknown.

Activated BCRs have also been shown to associate with c-Cbl/Cbl-b E3-ubiquitin ligases, which results in the ubiquitination of Igα and Igβ (80, 81). This is likely to play a role in both BCR-antigen complex internalization and downstream intracellular trafficking (82). Cbl-mediated ubiquitination can promote BCR uptake in an actin-dependent mechanism (83), providing a potential mechanism to resolve the binding competition between signaling and endocytic regulators. The molecular mechanisms of ubiquitin-driven BCR endocytosis remain uncharacterized. Recent proteomic analysis by Satpathy et al. confirmed PTM changes in numerous CCP components and actin regulators following BCR ligation (PICALM, SWAP70, DOCK8, EPS15, EPN2, WASH complex) (18). Additionally, several endocytic accessory proteins, including epsins and Eps15R, contain ubiquitin-binding domains which may mediate interaction with ubiquitinated BCR. Depletion of CIN85 or Cbl, whose role in BCR signaling is clear (84, 85), also inhibits FEME (24) and reduces Endophilin A2 recruitment to the BCR (23).

Cbl and Cbl-b promote antigen uptake in naïve but not GC B cells, despite their high expression in the latter (86). This may reflect the requirement for naïve B cells to efficiently internalize antigen irrespective of its affinity for the BCR (71) and future insight into the molecular mechanisms will be exciting.

### Altered BCR-Antigen Endocytosis in Malignant B Cells

**Figure 2:**
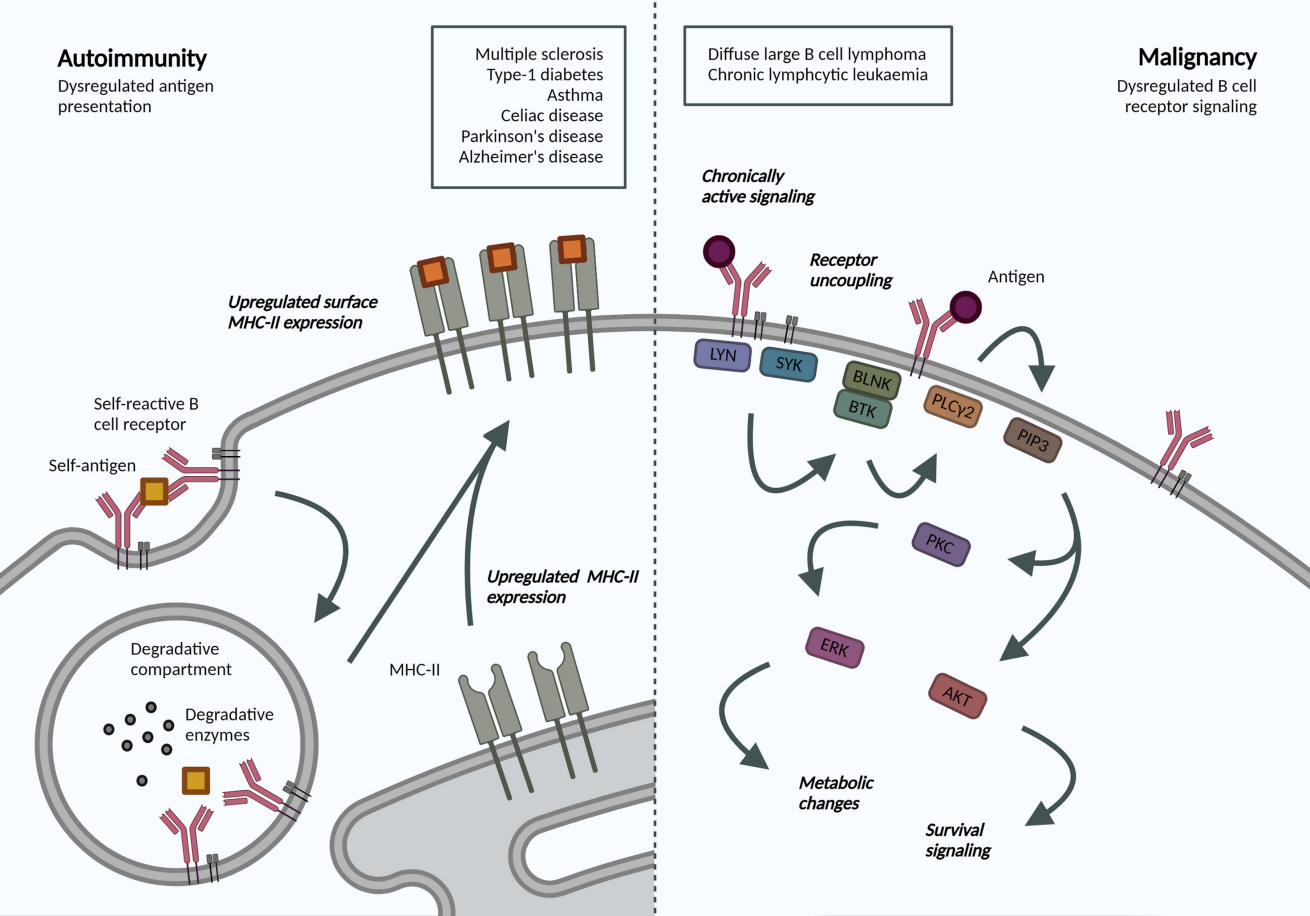
Altered BCR-antigen endocytosis, trafficking and presentation in B cell autoimmunity and malignancy. Autoimmunity. BCR-mediated self-antigen uptake can increase presentation of relevant peptides and drive disease progression. B cells in many autoimmune disorders exhibit increased surface MHC-II expression, though the repertoire of presented peptides has not been addressed. Malignancy. Following antigen binding to the BCR, a signaling cascade is initiated through phosphorylation of Igα/β ITAMs residues. Downstream activation of ERK and AKT mediates many metabolic changes and survival signaling, which are dysregulated in B cell malignancies due to localization and trafficking dynamics of the BCR. Created with BioRender.com.

The crucial interplay between BCR endocytosis and signaling is illustrated by the number of B cell malignancies that display altered antigen receptor endocytosis or trafficking (**Figure 2**). Igβ ITAM residues modulate ligand-induced signaling by regulating BCR internalization and are therefore essential for normal levels of cell surface BCR expression. Mutations in Igβ resulted in reduced BCR internalization and enhanced signaling in primary B cells, specifically Akt and Erk activation, which can promote cell survival and metabolic changes (87). Specific mutations in the first ITAM residue in Igβ have been observed in activated B cell-like (ABC) diffuse large B cell lymphoma (DLBCL). These increase surface BCR expression by attenuating Lyn kinase activity and reducing internalization, leading to chronic active BCR signaling as a pathogenic mechanism in ABC DLBCL (88).

Interestingly, in chronic lymphocytic leukemia (CLL), anergic cells exhibit reduced BCR signaling and increased internalization when compared to non-anergic counterparts (89). The specific signaling pathways and endocytic regulators were not addressed but the model supports mutually exclusive signaling and internalization functions and highlights changes in internalization dynamics in B cell malignancy. Understanding the dependency of different leukemias and lymphomas on distinct endocytic processes and how these may alter cell signaling can reveal novel disease mechanisms and therapeutic targets, specifically in our move towards precision medicine.

### B Cell Antigen Trafficking and Presentation in Autoimmunity

Antigen presentation by B cells has been associated with the development and progression of various autoimmune disorders. An antigen presentation role for B cells, independent of autoantibody production, has been established in multiple sclerosis (MS) (90). Indeed, surface MHC-II expression was increased in B cells from relapsing MS patients (91), and this increase may be mechanistically linked to Epstein-Barr virus (EBV) infection (92). In animal models, B cell antigen presentation was sufficient to drive neuro-inflammation (93). B cell antigen presentation is also implicated in the development and progression of type-1 diabetes, irrespective of their ability to produce secreted antibodies (94), as well as asthma (95) and celiac disease (96). Most recently, single cell sequencing of B cells from patients with Parkinson’s disease also revealed increased expression of MHC-II genes (97); while transient B cell depletion significantly reduced Alzheimer’s disease progression in murine models (98). While these studies do not address the dynamics or specificity, they highlight an increased capacity for antigen-presentation in disease-associated B cells. Understanding antigen trafficking and presentation would provide exciting opportunities for B-cell targeted therapeutics in these pathologies.

### Unanswered Questions and Future Milestones

How are BCR antigen internalization and signaling coordinated?

What is the fate of BCR-antigen complexes endocytosed by distinct mechanisms? How are these altered in malignancy, and can they promote progression?

How are actin and proteasome remodeling processes coordinated with endocytic mechanisms?

How are BCR uptake processes altered with ageing, and do they contribute to reduced B cell responses?

What is the role of ubiquitination in BCR antigen internalization? What is the relationship between different post-translational modifications and how do they interact with endocytic regulators?

## Concluding Remarks

Antigen-induced B cell activation plays a key role in the adaptive immune response by regulating BCR signaling capacity and antigen availability for presentation. Many questions remain and the field will benefit from advances in imaging, genetic and proteomics techniques. A better understanding of these processes could provide the tools to modulate the process with direct implications for vaccine design, and potential for novel targeted therapeutics in B cell malignancies and autoimmune diseases.

## Author Contributions

DM and ANMS wrote and edited the manuscript. All authors contributed to the article and approved the submitted version.

## Funding 

We would like to acknowledge funding from the NI Department for the Economy (ANMS), QUB Patrick G Johnston fellowship (DM), Royal Society (RGS\R2\212016) (DM) and Medical Research Council (MR/W025868/1) (DM).

## Conflict of Interest

The authors declare that the research was conducted in the absence of any commercial or financial relationships that could be construed as a potential conflict of interest.

## Publisher’s Note

All claims expressed in this article are solely those of the authors and do not necessarily represent those of their affiliated organizations, or those of the publisher, the editors and the reviewers. Any product that may be evaluated in this article, or claim that may be made by its manufacturer, is not guaranteed or endorsed by the publisher.

## Glossary

**Table:**

APC	antigen presentation cell
BCR	B cell receptor
CCP	clathrin-coated pit
CCV	clathrin-coated vesicle
CLL	chronic lymphocytic leukemia
CME	clathrin-mediated endocytosis
DC	dendritic cell
DLBCL	diffuse large B cell lymphoma
EAE	experimental autoimmune encephalomyelitis
EBV	Esptein-Barr Virus
ESCRT	endosomal sorting complexes required for transport
FEME	fast endophilin-mediated endocytosis
GC	germinal center
GEF	guanine nucleotide exchange factor
GTP	guanosine triphosphate
HLA	human leukocyte antigen
ITAM	immunoreceptor tyrosine-based activation motif
MHC	major histocompatibility complex
MS	multiple sclerosis
NFκB	nuclear factor kappa B
NOD	non-obese diabetic
PICALM	phosphatidylinositol binding clathrin assembly protein
PM	plasma membrane
PTM	post-translational modification
SNX	sorting nexin
WASH	Wiskott-Aldrich Syndrome protein and scar homologue complex
WASp	Wiskott-Aldrich Syndrome protein
